# Cardiovascular risk and kidney function profiling using conventional and novel biomarkers in young adults: the African-PREDICT study

**DOI:** 10.1186/s12882-023-03100-w

**Published:** 2023-04-13

**Authors:** A Degenaar, A Jacobs, R Kruger, C Delles, H Mischak, CMC Mels

**Affiliations:** 1 Hypertension in Africa Research Team (HART), North-West University, Potchefstroom, South Africa; 2 MRC Research Unit: Hypertension and Cardiovascular Disease, North-West University, Potchefstroom, South Africa; 3 Institute of Cardiovascular and Medical Sciences, University of Glasgow, Glasgow, UK; 4 Mosaiques Diagnostics GmbH, Hannover, Germany

**Keywords:** Age, Biomarkers, Cardiovascular risk factors, Kidney function

## Abstract

**Background:**

Low- and middle-income countries experience an increasing burden of chronic kidney disease. Cardiovascular risk factors, including advancing age, may contribute to this phenomenon. We (i) profiled cardiovascular risk factors and different biomarkers of subclinical kidney function and (ii) investigated the relationship between these variables.

**Methods:**

We cross-sectionally analysed 956 apparently healthy adults between 20 and 30 years of age. Cardiovascular risk factors such as high adiposity, blood pressure, glucose levels, adverse lipid profiles and lifestyle factors were measured. Various biomarkers were used to assess subclinical kidney function, including estimated glomerular filtration rate (eGFR), urinary albumin, uromodulin and the CKD273 urinary proteomics classifier. These biomarkers were used to divide the total population into quartiles to compare extremes (25^th^ percentiles) on the normal kidney function continuum. The lower 25^th^ percentiles of eGFR and uromodulin and the upper 25^th^ percentiles of urinary albumin and the CKD273 classifier represented the more unfavourable kidney function groups.

**Results:**

In the lower 25^th^ percentiles of eGFR and uromodulin and the upper 25^th^ percentile of the CKD273 classifier, more adverse cardiovascular profiles were observed. In multi-variable adjusted regression analyses performed in the total group, eGFR associated negatively with HDL-C (β= -0.44; p < 0.001) and GGT (β= -0.24; p < 0.001), while the CKD273 classifier associated positively with age and these same risk factors (age: β = 0.10; p = 0.021, HDL-C: β = 0.23; p < 0.001, GGT: β = 0.14; p = 0.002).

**Conclusion:**

Age, lifestyle and health measures impact kidney health even in the third decade.

**Supplementary Information:**

The online version contains supplementary material available at 10.1186/s12882-023-03100-w.

## Background

Chronic kidney disease (CKD) is becoming a major public health burden [1], especially in low- and middle-income countries [2, 3]. Although many factors may contribute to the exacerbation of CKD in these countries, the most significant factors are poor public healthcare systems and a high prevalence of cardiovascular risk factors such as hypertension, diabetes, obesity and advancing age [2–6]. Kidney function may already start to decline in young adults between 30 and 40 years of age [6, 7], possibly due to the increased prevalence of hypertension, diabetes and obesity in adults in this age range [4, 8].

Various biomarkers of kidney function, including conventional (estimated glomerular filtration rate (eGFR) and urinary albumin) and novel biomarkers (uromodulin (UMOD)), are used to diagnose kidney abnormalities and monitor kidney function [9, 10]. Recent advances in the field of proteomics have enabled the identification of relatively novel urinary biomarkers such as the CKD273 proteomics classifier [11, 12]. The CKD273 classifier has been found to yield better results than conventional biomarkers in predicting and diagnosing CKD, since it can identify subcellular changes in kidney function and predict the development of CKD at an early stage [12–14]. The use of such a cutting edge biomarker is not always feasible in a low-resource setting such as Africa due to the high cost of analysis and the necessary specialised equipment [15, 16]. Previous studies that have investigated the usefulness and efficiency of biomarkers of kidney function mainly focused on individuals with established CKD [17, 18] and older study populations [10, 18, 19].

Risk factors such as obesity, hypertension, diabetes, dyslipidaemia, low socio-economic status (SES), smoking, alcohol consumption and a sedentary lifestyle are associated with kidney dysfunction and the development of cardiovascular diseases [4, 5, 8, 20, 21]. Limited studies have focused on young adults and the use of different biomarkers of kidney function and the cardiovascular risk factors that play a role in early deterioration in kidney function. The aims of this study were to profile young adults (aged 20–30 years), stratified by degrees of kidney function based on biomarker levels, while comparing cardiovascular risk factors and subclinical kidney function biomarkers, and to explore the link between these variables.

## Methods

### Study population and organisational procedures

This study forms part of the larger African PRospective study on the Early Detection and Identification of Cardiovascular disease and hyperTension (African-PREDICT). The aim of the African-PREDICT study is to investigate early pathophysiological changes associated with cardiovascular disease development and to identify novel early markers or predictors of the development of cardiovascular disease by following young, healthy adults over a period of 10–20 years [22]. The African-PREDICT study is conducted at the North-West University (Potchefstroom campus), South Africa. Participants were recruited from Potchefstroom and the surrounding areas. Young black and white men and women between the ages of 20 and 30 years were initially screened and those who met the criteria were included in the study. The exclusion criteria were an office blood pressure > 140/90 mmHg, human immunodeficiency virus (HIV) infection, pregnancy, lactation, previous diagnosis of any chronic disease or chronic medication use, recent surgery or trauma (within the past three months), or previous history of cardiovascular disease. For this sub-study, baseline data from the African-PREDICT study were cross-sectionally analysed. Participants with missing data for biomarkers of kidney function (eGFR, urinary albumin, UMOD or the CKD273 classifier) were excluded (N = 306) resulting in a total of N = 956 participants.

All procedures were thoroughly explained to the participants and written informed consent was obtained before any measurements commenced. Data were collected and managed using the REDCap electronic data capture system [23].

### Questionnaires and general demographics

Participants completed a general demographic and health questionnaire with the assistance of trained researchers to obtain information regarding age, sex, ethnicity, smoking, alcohol intake, medication use and family history of cardiovascular disease. The SES of a participant was derived from three categories included in the general health questionnaire, namely skills level, education, and household income. Each category was awarded points and participants were then categorised into low, middle or high socio-economic groups [24].

### Anthropometric and physical activity measurements

The International Society for the Advancement of Kinanthropometry guidelines [25] were followed to measure height (SECA 213 Portable Stadiometer, SECA, Hamburg, Germany), weight (SECA 813 Electronic Scale, SECA, Hamburg, Germany) and waist circumference (Lufkin Steel Anthropometric Tape (W606PM); Lufkin, Apex, USA). Body mass index (BMI) and waist-to-height ratio (WHtR) were calculated. Participants were also fitted with an ActiHeart physical activity monitor (CamNtech Ltd., England, UK) that recorded total energy expenditure (TEE), which was indexed by weight and expressed as kCal/kg/day. The ActiHeart device was worn for a maximum of seven days.

### Cardiovascular measurements

The 2018 European Society of Cardiology (ESC) and European Society of Hypertension (ESH) Guidelines for the management of arterial hypertension were followed to measure blood pressure in this study [26]. Twenty-four-hour (24 h) blood pressure was measured using an ambulatory blood pressure monitoring (ABPM) device (Card(X)plore, Meditech, Budapest, Hungary). Ambulatory measurements included systolic blood pressure (SBP), diastolic blood pressure (DBP) and pulse pressure (PP). An appropriate-sized cuff was fitted to the participants’ non-dominant arm and participants were given instructions on how to ensure successful inflations across the 24 h period. The ABPM device was programmed so that it measured blood pressure in 30-minute intervals during the day (06:00–22:00) and in hourly intervals during the night (22:00–06:00). The mean successful inflation rate of the study population over a 24 h period was 88.2 ± 12.2%. Participants were also provided with an ambulatory diary card where they reported all activities that might influence blood pressure during the 24 h period.

Office brachial blood pressure was obtained by using the Dinamap Procare 100 Vital Signs Monitor (GE Medical Systems, Milwaukee, USA) and an appropriate-sized GE Critikon latex-free Dura-Cuff. Participants were requested not to have eaten, smoked, or exercised for at least 30 min before the measurements were performed. The first measurement was taken on the left arm after the participant had been in a resting state for five minutes (seated with the arm supported at heart level). Thereafter, blood pressure was taken in duplicate on the right arm and a final measurement was done on the left upper arm. There was a five-minute interval between the first two measurements and the duplicate measurements. The mean of all four blood pressure measurements were used and for each measurement the SBP, DBP, PP and heart rate were recorded.

### Blood sampling and biochemical analyses

Participants were asked to fast overnight and early morning blood samples were taken from the antebrachial vein branches by a registered research nurse using a sterile winged infusion set and syringes. Participants were also requested to provide a spot urine sample. After biological samples had been collected, all samples were taken to the on-site research laboratory to be prepared, aliquoted into cryovials and stored in bio-freezers (-80°C).

Basic serum analyses were performed for C-reactive protein (high-sensitivity), gamma-glutamyl transferase (GGT), albumin, creatinine, and lipids (high-density lipoprotein cholesterol (HDL-C), low-density lipoprotein cholesterol (LDL-C), total cholesterol, and triglycerides). The triglyceride-to-HDL-C ratio was also calculated. Glycated haemoglobin (HbA1c) was analysed in ethylenediaminetetraacetic acid (EDTA) whole blood samples and glucose levels were determined in sodium fluoride plasma samples. All of the above measurements were performed with the Cobas Integra 400 plus analyser (Roche, Basel, Switzerland). Serum cotinine was analysed by using a chemiluminescence method on the Immulite (Siemens, Erlangen, Germany) apparatus. Since the Chronic Kidney Disease Epidemiology Collaboration (CKD-EPI) equation that takes race into consideration has indicated a large bias in South African patients [27], we calculated eGFR by using the CKD-EPI creatinine formula that does not take race into consideration by using serum creatinine levels [28]. Spot urine samples were used to determine albumin and creatinine levels. The urinary UMOD concentration was measured by ELISA as previously described [29] with a standard curve generated from human UMOD (stock solution, 100 µg/ml; Millipore).

### Proteomics analyses

For urinary peptidomics, 700 µl of spot urine was diluted with 700 µl 2 M urea and 0.1 M ammonium hydroxide containing 0.02% sodium dodecyl sulphate. A 20 kDa molecular weight cut-off centrifugal ultrafiltration unit (Centrisart, Sarorius, Göttingen, Germany) was used for ultracentrifugation at 3000 x g for 1 h at 4 °C. The filtrate was then desalted to remove urea, electrolytes and salts with a PD-10 desalting column (Amersham Bioscience, Buckinghamshire, UK) and peptide elution was achieved with 0.01% aqueous ammonium hydroxide. Hereafter samples were lyophilised, stored at 4 °C, and re-suspended in high-performance liquid chromatography grade water to a final concentration of 2 µg/µl before analysis.

Capillary Electrophoresis Time of Flight Mass Spectrometry (CE-TOF-MS) was performed using a P/ACE MDQ capillary electrophoresis system (Beckman Coulter, Fullerton, USA) coupled with a Micro-TOF mass spectrometer (Bruker Daltonic, Bremen, Germany) as previously described [16]. Samples (250 nl) were injected with 2 psi for 99 s and separation of peptides in the cartridge (maintained at 25 °C) was achieved at 25 kV for 30 min followed by a 0.5 psi increase in pressure for another 35 min. The running buffer consisted of 79:20:1 (v/v) water, acetonitrile and formic acid, and the sheath liquid consisted of 30% isopropanol, 0.4% formic acid in high-performance liquid chromatography grade water. The electrospray ionisation sprayer (Agilent Technologies, California, USA) was grounded, and the ion spray inference potential was set at -4.5 kV. Mass spectra over a mass-to-charge ratio of 350–3000 were collected for every three seconds.

The Mosaiques Visu software [30] was used for peak picking, deconvolution and de-isotoping of mass spectral ion peaks. The capillary electrophoresis migration time and ion signal intensity were normalised based on the reference signal from internal peptide standards or calibrators (peptides from housekeeping proteins in rats) [31]. A local regression algorithm with calibrators was used for calibration. The generated peak list for each peptide comprised of the molecular weight (kDa), normalised capillary electrophoresis migration time (minutes) and normalised signal intensity.

The list of peptides from all the samples that met the quality control criteria was annotated and compared in a Microsoft Structured-Query Language database. The criteria applied to cluster peptides in different samples were (i) mass deviation < ± 50 ppm for peptides < 800 Da with a gradual increase to ± 75 ppm for larger peptides (20 kDa); (ii) capillary electrophoresis migration time deviation with a linear increase from ± 0.4 min to ± 2.5 min in the range from 19 to 50 min. A unique identification number was given to each peptide. The validated urinary proteomics classifier scores for chronic kidney disease (CKD273) were calculated [11, 16, 32].

### Statistical analyses

Statistical analyses were performed with IBM® SPSS® Statistics version 27 software (IBM Corporation; Armonk, New York, USA). GraphPad Prism version 5.03 (GraphPad Software Inc., CA, USA) was used for the graphical illustrations of the data. Variables were tested for normality by using Q-Q plots and logarithmically transformed if skewed. Log-transformed variables included total cholesterol, HDL-C, LDL-C, triglycerides, triglyceride-to-HDL-C ratio, C-reactive protein, GGT, cotinine, urinary albumin and UMOD. Continuous data with a normal distribution were reported as the arithmetic mean and standard deviation, logarithmically transformed variables were presented by the geometric mean and 5th and 95th percentile intervals and categorical data as proportions.

Although our study population consisted of young, apparently healthy adults with normal kidney function, we divided biomarkers of kidney function in lower and upper 25^th^ percentiles to investigate outliers in the normal continuum. The lower 25^th^ percentiles of eGFR and UMOD and the upper 25^th^ percentiles of urinary albumin and the CKD273 classifier were regarded as the groups with more unfavourable kidney function (although not pathological). Profiling of cardiovascular risk factors and kidney function biomarkers between lower and upper 25^th^ percentiles of novel and conventional kidney function biomarkers were done by using independent T-tests for continuous variables and Chi-square tests for categorical variables. In addition, an analysis of covariance was used to adjust for urinary creatinine in the urinary albumin comparison and when comparing cardiovascular risk factors between the lower and upper 25^th^ percentiles of urinary albumin.

Multiple regression analyses were used to determine which cardiovascular risk factors (age, adiposity, blood pressure, glucose level, lipid profile, lifestyle factors) contributed to the variance of each kidney function biomarker (eGFR, urinary albumin, UMOD and the CKD273 classifier). Based on the literature and exploratory bivariate correlations, covariates entered into the models were age, sex, ethnicity, waist circumference, bSBP, HbA1c, HDL-C, SES score, GGT, cotinine and TEE. A sensitivity analysis was performed by adding C-reactive protein as a covariate in all the multiple regression models to determine whether a marker of inflammation contributed to the variance in each of the kidney function biomarkers. Partial correlations were used to determine how biomarkers of kidney function (eGFR, urinary albumin, UMOD and the CKD273 classifier) associated with one another, while taking age, sex, and ethnicity into account.

Given that age is one of the main cardiovascular risk factors in our study, we used the CKD-EPI creatinine formula without taking age into consideration. In addition, urinary creatinine was included as a covariate for models with urinary albumin as a dependent variable. In this study, a p-value < 0.05 was considered statistically significant. Furthermore, given the large number of tests performed, we adjusted for multiple testing by using the Benjamini-Hochberg procedure.

## Results

The descriptive characteristics of the study population stratified by degrees of kidney function (based on eGFR levels) are presented in Table 1. In the lowest quartile of eGFR (indicative of more unfavourable kidney function) an adverse cardiovascular profile was observed when compared to the highest eGFR quartile. This included higher age, total cholesterol, LDL-C, HDL-C, triglycerides, GGT levels as well as SES-scores and lower TEE (all p < 0.05). In addition, higher CKD273 classifier levels were present in the lowest quartile of eGFR (p < 0.001). When comparing cardiovascular risk factors between lower and upper 25^th^ percentiles of other kidney function biomarkers (urinary albumin, UMOD and the CKD273 classifier) more adverse cardiovascular profiles were also present in the lower 25^th^ percentile of UMOD and the upper 25^th^ percentile of the CKD273 classifier (See Supplementary Table 1, Additional File 1). However, the risk factors did not differ between the lower and upper percentiles of urinary albumin. With regards to the biomarkers, lower eGFR levels were present in the lower 25^th^ percentile of urinary albumin and the upper 25^th^ percentile of the CKD273 classifier.

**Fig. 1:**
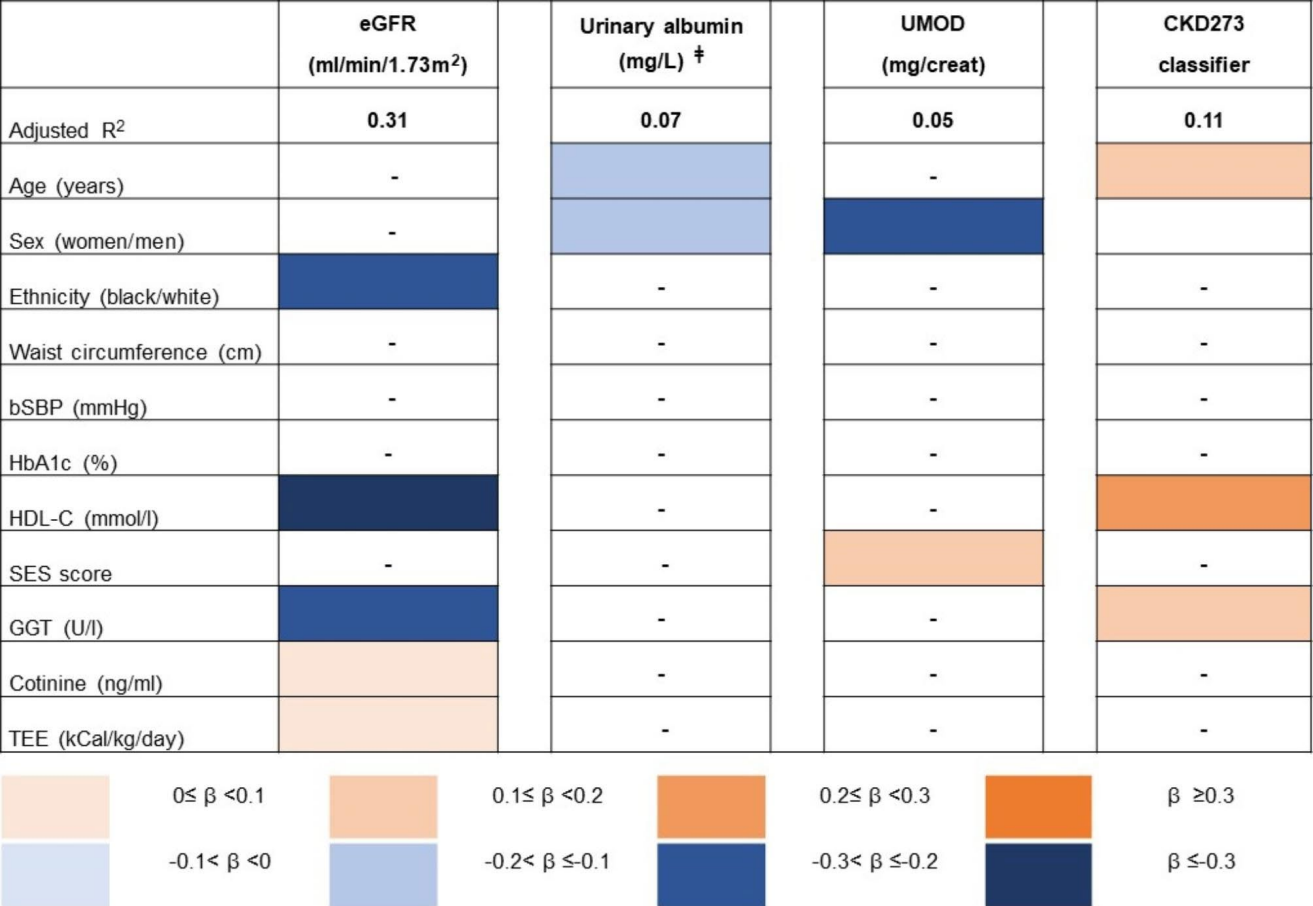
Multiple regression analyses of kidney function biomarkers and cardiovascular risk factors in the total group. Risk factors that significantly associated with the kidney function biomarkers are indicated in colour (p < 0.05). A darker shade of colour indicates a higher β value. Abbreviations: eGFR, estimated glomerular filtration rate; UMOD, uromodulin; bSBP, brachial systolic blood pressure; HbA1c, glycated haemoglobin; HDL-C, high-density lipoprotein cholesterol; SES, socio-economic status; GGT, gamma-glutamyl transferase; TEE, total energy expenditure. (^‡^ Urinary albumin additionally adjusted for urinary creatinine. - Denotes variables that did not contribute to the different models).

Aligned with our aim we determined whether cardiovascular risk factors were associated with different subclinical kidney function biomarkers (Fig. 1, See Supplementary Table 2, Additional File 1). After multiple regression analyses, we observed the following associations. In the total group, eGFR was negatively associated with HDL-C (β= -0.44; p < 0.001) and GGT (β= -0.24; p < 0.001) and positively associated with cotinine (β = 0.08; p = 0.031) and TEE (β = 0.08; p = 0.026). In addition, eGFR associated with a white ethnicity (β= -0.23; p < 0.001). Albumin, on the other hand, was negatively associated with age (β= -0.09; p = 0.031) and women (β= -0.09; p = 0.032), while UMOD was positively associated with SES score (β = 0.09; p = 0.038) and men (β= -0.22; p < 0.001). Furthermore, the CKD273 classifier associated positively with age (β = 0.10; p = 0.021), HDL-C (β = 0.23; p < 0.001) and GGT (β = 0.14; p = 0.002).

**Table 1:** Characteristics of the study population stratified according to kidney function (based on eGFR levels)

	Total group	Quartile 1 (eGFR < 131 ml/min/1.73m^2^)	Quartile 2 (eGFR 131–145 ml/min/1.73m^2^)	Quartile 3 (eGFR 146–155 ml/min/1.73m^2^)	Quartile 4 (eGFR ≥ 156 ml/min/1.73m^2^)	
N	956	239	239	239	239	
Age (years)	24.5 ± 3.12	25.5 ± 2.78^bc^	25.4 ± 2.99^de^	23.6 ± 2.90^bd^	23.4 ± 3.17^ce^	**< 0.001**
Ethnicity (black, %)	472 (49.4)	88 (36.8)	129 (54.0)	134 (56.1)	121 (50.6)	**< 0.001**
Sex (women, %)	476 (49.8)	129 (54.0)	120 (50.2)	127 (53.1)	100 (41.8)	**0.007**
Body mass index (kg/m^2^)	25.1 ± 5.50	25.6 ± 4.88	25.6 ± 6.64	25.0 ± 6.15	24.1 ± 5.19	0.055
Waist circumference (cm)	80.5 ± 12.5	81.6 ± 12.1	81.6 ± 13.1	79.7 ± 13.2	79.1 ± 11.1	0.291
Waist-to-height ratio	0.48 ± 0.07	0.48 ± 0.07	0.49 ± 0.07^e^	0.48 ± 0.08	0.47 ± 0.06^e^	0.512
**Blood pressure measurements**						
24 h SBP (mmHg)	117 ± 9.58	117 ± 9.88	117 ± 9.56	116 ± 9.63	118 ± 9.28	0.648
24 h DBP (mmHg)	69 ± 5.96	69 ± 6.12	69 ± 5.91	68 ± 5.91	68 ± 5.87	0.515
24 h PP (mmHg)	49 ± 7.24	48 ± 7.60	48 ± 6.62	48 ± 7.63	49 ± 7.05	0.264
Office SBP (mmHg)	123 ± 10.5	124 ± 11.4	123 ± 11.1	122 ± 9.55	122 ± 9.58	0.181
Office DBP (mmHg)	73 ± 7.87	74 ± 8.49	74 ± 7.89	74 ± 7.41	72 ± 7.65	0.653
Office PP (mmHg)	49 ± 7.82	50 ± 8.51	49 ± 7.35	48 ± 7.54	49 ± 7.81	0.226
**Biochemical markers**						
Total cholesterol (mmol/l)	3.46 (1.95; 5.78)	4.36 (2.87; 6.11)^abc^	3.91 (2.65; 5.82)^ade^	3.33 (2.11; 5.54)^bdf^	2.53 (1.59; 4.02)^cef^	**< 0.001**
HDL-C (mmol/l)	1.04 (0.55; 1.87)	1.31 (0.80; 2.24)^abc^	1.13 (0.65; 1.84) ^ade^	1.00 (0.54; 1.71)^bdf^	0.80 (0.50; 1.41)^cef^	**< 0.001**
LDL-C (mmol/l)	2.19 (1.04; 4.18)	2.69 (1.46; 4.51)^bc^	2.53 (1.45; 4.16)^de^	2.18 (1.11; 4.11)^bdf^	1.55 (0.82; 2.88)^cef^	**< 0.001**
Triglycerides (mmol/l)	0.70 (0.30; 1.82)	0.88 (0.46; 1.93)^bc^	0.83 (0.35; 2.45)^de^	0.66 (0.32; 1.62)^bdf^	0.50 (0.26; 1.09)^cef^	**< 0.001**
Triglyceride-to-HDL-C ratio	0.67 (0.28; 2.09)	0.67 (0.29; 1.90)	0.73 (0.27; 3.27)^e^	0.66 (0.29; 2.09)	0.63 (0.26; 1.76)^e^	0.264
HbA1c (%)	5.31 ± 0.32	5.32 ± 0.27	5.36 ± 0.31^e^	5.30 ± 0.33	5.26 ± 0.33^e^	0.198
C-reactive protein (mg/l)	0.83 (0.07; 9.28)	0.97 (0.08; 10.9)^c^	0.97 (0.13; 8.74)^e^	0.98 (0.10; 10.2)^f^	0.53 (0.04; 9.31)^cef^	**< 0.001**
GGT(U/l)	17.8 (5.80; 54.7)	21.3 (9.00; 54.7)^bc^	21.8 (7.80; 66.1)^de^	17.1 (5.70; 52.4)^bdf^	12.5 (4.80; 42.5)^cef^	**< 0.001**
Cotinine (ng/ml)	3.63 (1.00; 327)	2.74 (1; 291)	4.40 (1.00; 384)	3.92 (1.00; 310)	3.73 (1.00; 339)	0.676
Creatinine (µmol/l)	65.5 ± 17.6	85.6 ± 14.5 ^abc^	69.9 ± 9.06 ^ade^	59.8 ± 8.27 ^bdf^	46.8 ± 8.85 ^cef^	**< 0.001**
**Kidney function markers**						
eGFR (ml/min/1.73m^2^)	143 ± 21.9	113 ± 13.4 ^abc^	141 ± 1.49 ^ade^	151 ± 2.92 ^bdf^	169 ± 9.31 ^cef^	**< 0.001**
Urinary albumin (mg/L)^#^	5.43 (5.21; 5.65)	5.31 (4.90; 5.75)	5.30 (4.89; 5.74)	5.40 (4.98; 5.85)	5.71 (5.27; 6.18)	0.706
UMOD (mg/creat)	40.9 (18.9; 81.3)	39.3 (15.6; 84.5)	42.0 (20.0; 86.4)	38.8 (14.6; 82.1)	43.5 (23.7; 79.8)	0.071
CKD273 classifier	-0.55 ± 0.39	− 0.45 ± 0.36^bc^	-0.46 ± 0.39^de^	-0.58 ± 0.40^bdf^	− 0.72 ± 0.36^cef^	**< 0.001**
**Lifestyle factors**						
SES score	20.5 ± 6.03	22.9 ± 5.96^abc^	20.8 ± 6.50^ad^	19.0 ± 5.40^bd^	19.4 ± 5.44^c^	**< 0.001**
SR smoking, n (%)	233 (24.4)	44 (18.4)	66 (27.6)	59 (24.7)	64 (26.9)	0.136
SR alcohol use, n (%)	515 (54.2)	127 (53.6)	130 (54.9)	131 (54.8)	127 (53.6)	0.999
TEE (kCal/kg/day)	32.7 ± 9.18	31.1 ± 4.46^bc^	33.3 ± 16.9	32.9 ± 4.22^b^	33.5 ± 4.75^c^	**< 0.001**

Abbreviations: eGFR, estimated glomerular filtration rate; SBP, systolic blood pressure; DBP, diastolic blood pressure; PP, pulse pressure; HDL-C, high-density lipoprotein cholesterol; LDL-C, low-density lipoprotein cholesterol; HbA1c, glycated haemoglobin; GGT, gamma-glutamyl transferase; UMOD, uromodulin; SES, socio-economic status; SR, self-reported; TEE, total energy expenditure.

Notes: values are expressed as arithmetic mean ± standard deviation, geometric mean with 5th and 95th percentiles, or frequency and percentage.

^**#**^ Urinary albumin ANCOVA (adjusted for urinary creatinine) expressed as least square mean with confidence intervals.

^a^ Statistical significance between quartile 1 and 2; ^b^ statistical significance between quartile 1 and 3; ^c^ statistical significance between quartile 1 and 4; ^d^ statistical significance between quartile 2 and 3; ^e^ statistical significance between quartile 2 and 4; ^f^ statistical significance between quartile 3 and 4.

We performed partial correlations while adjusting for age, sex, and ethnicity to determine how the other biomarkers of kidney function associate with eGFR (See Supplementary Table 3, Additional File 1). In the total group, eGFR associated positively with UMOD (p = 0.002) and negatively with the CKD273 classifier (p < 0.001).

In a sensitivity analysis, where the multiple regression analyses were repeated after adding C-reactive protein as an additional risk factor into the models, similar results were observed.

## Discussion

In this study we aimed to (i) profile cardiovascular risk factors and kidney function in young adults by using various biomarkers of subclinical kidney function and (ii) determined whether these risk factors are related to different subclinical measures of kidney function. Since our study population included young and apparently healthy adults with normal kidney function, we divided all the biomarkers of kidney function into quartiles and used the lower and upper 25^th^ percentiles to investigate extremes in the normal continuum. The lower 25^th^ percentiles of eGFR and UMOD and the upper 25^th^ percentiles of urinary albumin and the CKD273 classifier represented the more unfavourable kidney function groups.

When comparing cardiovascular profiles between the lower and upper 25^th^ percentiles of the different kidney function biomarkers, several risk factors including higher age, adiposity, blood pressure, GGT, an adverse lipid profile (high total cholesterol, HDL-C, LDL-C, triglycerides and triglyceride-to-HDL-C ratio) and detrimental lifestyle factors were present in the more unfavourable eGFR, UMOD and CKD273 classifier groups. Interestingly, at this young age (20–30 years), age associated positively with the CKD273 classifier while other cardiovascular risk factors such as HDL-C and GGT only associated with eGFR (negative associations) and the CKD273 classifier (positive associations).

It is well known that obesity, hypertension and dyslipidaemia are associated with a decline in kidney function [5, 20]. These cardiovascular risk factors contribute to kidney damage through various mechanisms, including increased insulin resistance, inflammation, oxidative stress and renin-angiotensin-aldosterone system activation, which in turn can promote the development of kidney damage in young adults [8, 33–35]. Therefore, the higher levels of adiposity, blood pressure and adverse lipid profiles in some of the more unfavourable kidney function groups in our study are expected. However, the levels of these cardiovascular risk factors were only slightly higher and therefore still within the normal reference ranges (not clinically regarded as pathological) [36–38].

We found a positive association between the CKD273 classifier and advancing age (even in young adults). Recently, gradual increases in the CKD273 classifier score have been associated with a decline in kidney function, which enables the early detection of disease onset [13, 14]. During the ageing process, the kidneys undergo various complex structural and functional changes that in turn ultimately lead to kidney pathology [6]. Advancing age is characterised by a progressive decrease in kidney volume and increased focal and global glomerulosclerosis, arteriolosclerosis and interstitial fibrosis [6, 39]. In addition, a loss of peritubular capillaries has been observed during kidney ageing and is strongly associated with interstitial fibrosis and may predict kidney function decline [6]. Fibrosis is a central element in all forms of CKD and has recently been suggested to be involved in the early stages of disease onset [19]. Thus, the association between the CKD273 classifier and the decline in kidney function at a young age may be explained by the increase in fibrosis, since most of the peptides in the classifier are indicative of dysregulated collagen metabolism [14].

In this study there were independent associations of eGFR (negative association) and the CKD273 classifier (positive association) with GGT. Previous studies have indicated that elevated levels of GGT are associated with an increased risk of CKD and end-stage renal disease development [40, 41]. In addition, elevated GGT levels have been suggested to be an early and sensitive marker of oxidative stress [42]. Our finding suggests that oxidative stress may play an important role in the deterioration of kidney function at an early age. Future studies are needed to explore the association between GGT, kidney function deterioration and the mechanisms through which GGT may promote kidney damage in young adults.

In addition, we also indicated associations between eGFR (negative association) and the CKD273 classifier (positive association) with HDL-C. Similar findings were reported in middle-aged (58 ± 3.8 years) nondiabetic individuals with pre-existing CKD, in which elevated HDL-C levels were associated with an accelerated decline in eGFR [43]. These results are further supported by studies in the general population [44] and in individuals with nondialysis CKD [45], that indicate a U-shaped relationship between HDL-C and the progression of kidney disease. Therefore, even though low levels of HDL-C are traditionally considered to be a risk factor for a decline in kidney function [34], our results support the notion of a U-shaped relationship between HDL-C and the progression of kidney disease.

Although there are several biomarkers of kidney function, each biomarker can indicate kidney damage at distinct parts of the nephron [9, 46–48]. Conventional biomarkers such as eGFR and urinary albumin primarily indicate glomerular damage [9, 48], while novel biomarkers such as UMOD are very sensitive to kidney tubule damage [46, 47]. Therefore, even though urinary albumin and UMOD did not associate with many cardiovascular risk factors or kidney damage (as indicated by lower eGFR) in this study, these biomarkers may still be useful to monitor kidney damage. As our study population consisted of apparently healthy adults, it may also be possible that no kidney tubule damage is present. This in turn may explain why biomarkers such UMOD did not associate with kidney damage in our study population.

This study should be interpreted within the context of its strengths and limitations. This was a cross-sectional study to investigate cardiovascular risk factors and kidney function in young adults and therefore we cannot infer causality. Our findings cannot be generalised to the entire South African population, as we only included participants from the Potchefstroom and surrounding areas in the North-West province. Since we also excluded participants with any chronic diseases, our findings cannot be directly compared to individuals with CKD. Our study was well planned and executed under strictly controlled conditions in a well-equipped research facility. The study population included young and apparently healthy adults and provides information on early kidney function deterioration and cardiovascular risk factors, all of which may influence kidney disease development in young adults.

## Conclusion

In young adults between 20 and 30 years of age, various cardiovascular risk factors including age, lifestyle and health measures already impact kidney health.

## Electronic supplementary material

Below is the link to the electronic supplementary material.

Supplementary Material 1: **Table 1**: Cardiovascular profiles according to urinary albumin, UMOD and the CKD273 classifier as biomarkers of kidney function; **Table 2**: Multiple regression analyses of kidney function biomarkers and cardiovascular risk factors in the total group; **Table 3**: Partial correlations between different biomarkers of kidney function.

## Data Availability

The datasets generated and/or analysed during the current study are not publicly available due [It involves more privacy of participants] but are available from the corresponding author on reasonable request.

## Abbreviations

24 h: Twenty-four hour
ABPM: Ambulatory blood pressure monitoring
African-PREDICT: African PRospective study on the Early Detection and Identification of Cardiovascular disease and hyperTension
BMI: Body mass index
CE-TOF-MS: Capillary Electrophoresis Time of Flight Mass Spectrometry
CKD: Chronic kidney disease
CKD-EPI: Chronic Kidney Disease Epidemiology Collaboration
DBP: Diastolic blood pressure
EDTA: Ethylenediaminetetraacetic acid
eGFR: Estimated glomerular filtration rate
ESC: European Society of Cardiology
ESH: European Society of Hypertension
GGT: Gamma glutamyl-transferase
HbA1c: Glycated haemoglobin
HDL-C: High-density lipoprotein cholesterol
HIV: Human immunodeficiency virus
LDL-C: Low density-lipoprotein cholesterol
PP: Pulse pressure
SR: Self-reported
SBP: Systolic blood pressure
SES: Socioeconomic status
TEE: Total energy expenditure
UMOD: Uromodulin
WHtR: Waist-to-height ratio
