# Supplementary material for: Cardiovascular risk and kidney function profiling using conventional and novel biomarkers in young adults: the African-PREDICT study
Source: BMC Nephrol. 2023 Apr 13;24:96. doi: 10.1186/s12882-023-03100-w (PMC10103421; doi:10.1186/s12882-023-03100-w)
Supplement: Supplementary file 1 — Supplementary Material 1: Table 1: Cardiovascular profiles according to urinary albumin, UMOD and the CKD273 classifier as biomarkers of kidney function; Table 2: Multiple regression analyses of kidney function biomarkers and cardiovascular risk factors in the total group; Table 3: Partial correlations between different biomarkers of kidney function. [file 12882_2023_3100_MOESM1_ESM.docx]

## Supplementary tables

Supplementary Table 1: Cardiovascular profiles according to urinary albumin, UMOD and the CKD273 classifier as biomarkers of kidney function

|  | Urinary albumin ^ǂ^ | | UMOD | | | CKD273 classifier | |
| --- | --- | --- | --- | --- | --- | --- | --- |
|  | Lower 25^th^ percentile  (< 3.84 mg/L) | Upper 25^th^  percentile  (≥ 6.00 mg/L) | Lower 25^th^  percentile  (< 31.8 mg/creat) | | Upper 25^th^  percentile  (≥ 54.3 mg/creat) | Lower 25^th^ percentile  (< -0.83) | Upper 25^th^  percentile  (≥ -0.27) |
| N | 229 | 229 | 237 | | 237 | 244 | 240 |
| Age (years) | 25.2 ± 0.20 | 24.3 ± 0.21 * | 24.5 ± 3.10 | | 24.8 ± 3.08 | 23.9 ± 3.21 | 25.1 ± 2.95 * |
| Ethnicity (black, %) | 120 (52.4) | 115 (50.2) * | 119 (50.2) | | 106 (44.7) | 97 (39.8) | 123 (52.1) * |
| Sex (women, %) | 123 (53.7) | 120 (52.4) | 89 (37.6) | | 169 (71.3) * | 112 (45.9) | 126 (53.4) |
| Body mass index (kg/m^2^) | 25.5 ± 0.6 | 24.6 ± 0.37 | 24.8 ± 4.69 | | 25.4 ± 6.30 | 24.7 ± 5.07 | 26.0 ± 6.37 |
| Waist circumference (cm) | 80.7 ± 0.81 | 79.0 ± 0.83 | 80.7 ± 11.3 | | 79.7 ± 13.6 | 80.4 ± 11.8 | 82.3 ± 14.1 |
| Waist-to-height ratio | 0.48 ± 0.01 | 0.47 ± 0.01 | 0.47 ± 0.06 | | 0.48 ± 0.08 | 0.47 ± 0.07 | 0.49 ± 0.08 * |
| *Blood pressure measurements* | | | |  |  |  |  |
| 24h SBP (mmHg) | 116 ± 0.63 | 117 ± 0.65 | 118 ± 9.24 | | 115 ± 9.29 * | 118 ± 10.2 | 118 ±9.24 |
| 24h DBP (mmHg) | 69 ± 0.39 | 69 ± 0.41 | 69 ± 5.98 | | 69 ± 5.83 | 68 ± 5.78 | 69 ± 6.11 * |
| 24h PP (mmHg) | 48 ± 0.48 | 48 ± 0.49 | 49 ± 7.75 | | 47 ± 6.61 * | 50 ± 7.96 | 48 ± 6.51 * |
| Office SBP (mmHg) | 123 ± 0.68 | 123 ± 0.70 | 123 ± 10.0 | | 121 ± 10.5 * | 121 ± 9.80 | 125 ± 10.6 * |
| Office DBP (mmHg) | 74 ± 0.52 | 74 ± 0.53 | 73 ± 7.97 | | 73 ± 7.80 | 72 ± 7.43 | 75 ± 8.17 * |
| Office PP (mmHg) | 49 ± 0.51 | 49 ± 0.63 | 50 ± 8.52 | | 48 ± 7.18 * | 49 ± 7.37 | 50 ± 7.65 |
| *Biochemical markers* | | | |  |  |  |  |
| Total cholesterol (mmol/l) | 3.67 (3.52; 3.83) | 3.48 (3.33; 3.63) | 3.44 (1.96; 5.83) | | 3.64 (2.03; 5.80) | 3.03 (1.92; 5.25) | 3.98 (2.23; 5.95) * |
| HDL-C (mmol/l) | 1.09 (1.04; 1.14) | 1.08 (1.03; 1.14) | 1.03 (0.58; 1.81) | | 1.10 (0.58; 1.93) | 0.89 (0.51; 1.71) | 1.17 (0.66; 1.89) * |
| LDL-C (mmol/l) | 2.32 (2.20; 2.45) | 2.12 (2.06; 2.30) | 2.20 (1.03; 4.32) | | 2.28 (1.13; 4.11) | 1.93 (1.05; 3.93) | 2.55 (1.16; 4.40) * |
| Triglycerides (mmol/l) | 0.71 (0.66; 0.76) | 0.70 (0.65; 0.75) | 0.71 (0.32; 2.19) | | 0.68 (0.30; 1.66) | 0.62 (0.28; 1.52) | 0.82 (0.35; 2.19) * |
| Triglyceride-to-HDL-C ratio | 0.66 (0.60; 0.71) | 0.64 (0.59; 0.70) | 0.69 (0.27; 2.73) | | 0.62 (0.27; 1.67) | 0.70 (0.28; 2.23) | 0.70 (0.29; 2.50) |
| HbA1c (%) | 5.31 ± 0.02 | 5.33 ± 0.02 | 5.32 ± 0.30 | | 5.33 ± 0.32 | 5.28 ± 0.34 | 5.36 ± 0.30 * |
| C-reactive protein (mg/l) | 0.90 (0.75; 1.09) | 0.80 (0.66; 0.97) | 0.75 (0.07; 8.18) | | 1.09 (0.09; 11.9) * | 0.65 (0.05; 6.87) | 1.07 (0.10; 10.8) * |
| GGT(U/l) | 18.5 (16.9; 20.1) | 18.0 (16.4; 19.7) | 18.8 (5.90; 62.6) | | 16.6 (5.20; 48.0) | 14.4 (5.00; 44.3) | 21.5 (7.42; 65.6) * |
| Cotinine (ng/ml) | 3..07 (2.29; 4.11) | 3.67 (2.71; 4.98) | 3.70 (1.00; 422) | | 3.15 (1.00; 281) | 3.37 (1.00; 314) | 3.60 (1.00; 358) |
| Creatinine (µmol/l) | 66.8 (64.6; 69.0) | 61.8 (59.7; 64.0) * | 68.6 ± 18.3 | | 60.4 ± 16.5 * | 60.4 ± 17.3 | 70.6 ± 17.8 * |
| *Kidney function markers* | | | |  |  |  |  |
| eGFR (ml/min/1.73m^2^) | 137 ± 1.38 | 144 ± 1.41 * | 142 ± 22.3 | | 146 ± 20.3 | 151 ± 21.0 | 137 ± 20.9 * |
| Urinary albumin (mg/L) | 3.24 (3.16; 3.31) | 7.85 (7.67; 8.04) * | 5.47 (5.05; 5.93) | | 5.13 (4.73; 5.55) | 5.19 (4.80; 5.61) | 5.41 (4.98; 5.86) |
| UMOD (mg/creat) | 42.4 (40.0; 45.0) | 43.6 (41.0; 46.2) | 22.7 (10.1; 31.4) | | 69.9 (55.2; 99.7) * | 41.0 (22.2; 78.3) | 40.1 (17.0; 86.8) |
| CKD273 classifier | - 0.52 ± 0.03 | - 0.52 ± 0.03 | - 0.54 ± 0.41 | | - 0.53 ± 0.40 | -1.03 ± 0.16 | -0.03 ± 0.17 * |
| *Lifestyle factors* | | | |  |  |  |  |
| SES score | 21.4 ± 0.40 | 19.5 ± 0.40 * | 20.3 ± 6.20 | | 21.6 ±5.91 * | 20.4 ± 5.80 | 20.7 ± 6.71 |
| SR smoking, n (%) | 50 (21.9) | 52 (22.7) | 55 (23.2) | | 48 (20.3) | 60 (24.6) | 50 (21.3) |
| SR alcohol use, n (%) | 118 (52.4) | 134 (58.8) | 129 (54.7) | | 136 (57.4) | 127 (52.3) | 136 (58.1) |
| TEE (kCal/kg/day) | 32.1 ± 0.69 | 33.9 ± 0.75 | 32.6 ± 4.66 | | 32.2 ± 4.51 | 32.4 ± 4.28 | 32.5 ± 4.60 |

Abbreviations: eGFR, estimated glomerular filtration rate; SBP, systolic blood pressure; DBP, diastolic blood pressure; PP, pulse pressure; HDL-C, high-density lipoprotein cholesterol; LDL-C, low-density lipoprotein cholesterol; HbA1c, glycated haemoglobin; GGT, gamma-glutamyl transferase; eGFR, estimated glomerular filtration rate; UMOD, uromodulin; SES, socio-economic status; SR, self-reported; TEE, total energy expenditure.

Notes: values are expressed as arithmetic mean ± standard deviation, geometric mean with 5^th^ and 95^th^ percentiles, or frequency and percentage.

**^ǂ^** Risk factors in the lower and upper percentiles of urinary albumin adjusted for urinary creatinine (ANCOVA), values are presented as adjusted mean ± standard error, least quare mean with confidence intervals, or frequency and percentage.

* Statistical significance between the lower and upper 25^th^ percentiles

**Supplementary Table 2:** Multiple regression analyses of kidney function biomarkers and cardiovascular risk factors in the total group

|  | **eGFR (ml/min/1.73m^2^)** | **Urinary albumin  (mg/L) ^ǂ^** | **UMOD  (mg/creat)** | **CKD273 classifier** |
| --- | --- | --- | --- | --- |
| Adjusted R^2^ | 0.31 (p<0.001) | 0.07 (p<0.001) | 0.05 (p<0.001) | 0.11 (p<0.001) |
|  | β (95% CI) | β (95% CI) | β (95% CI) | β (95% CI) |
| Age (years) | **̶** | -0.09 (-0.16; -0.02) ^a^ | **̶** | 0.10 (0.03; 0.17) ^a^ |
| Sex (women/men) | **̶** | -0.09 (-0.33; -0.04) ^a^ | -0.22 (-0.58; -0.30) ^b^ | **̶** |
| Ethnicity (black/white) | -0.23 (-0.58; -0.32) ^b^ | **̶** | **̶** | **̶** |
| Waist circumference (cm) | **̶** | **̶** | **̶** | **̶** |
| bSBP (mmHg) | **̶** | **̶** | **̶** | **̶** |
| HbA1c (%) | **̶** | **̶** | **̶** | **̶** |
| HDL-C (mmol/l) | -0.44 (-0.50; -0.37) ^b^ | **̶** | **̶** | 0.23 (0.16; 0.30)^b^ |
| SES score | **̶** | **̶** | 0.09 (0.003; 0.03) ^a^ | **̶** |
| GGT (U/l) | -0.24 (-0.31; -0.17) ^b^ | **̶** | **̶** | 0.14 (0.06; 0.21) ^a^ |
| Cotinine (ng/ml) | 0.08 (0.02; 0.14) ^a^ | **̶** | **̶** | **̶** |
| TEE (kCal/kg/day) | 0.08 (0.02; 0.14) ^a^ | **̶** | **̶** | **̶** |

Variables included in the model: age, sex, ethnicity, waist circumference, bSBP, HbA1c, HDL-C, SES, GGT, cotinine and TEE.

**^ǂ^** Urinary albumin additionally adjusted for urinary creatinine.

Abbreviations: eGFR, estimated glomerular filtration rate; UMOD, uromodulin; bSBP, brachial systolic blood pressure; HbA1c, glycated haemoglobin; HDL-C, high-density lipoprotein cholesterol; SES, socio-economic status; GGT, gamma-glutamyl transferase; TEE, total energy expenditure.

**̶** Denotes variables that did not contribute to the different models.

^a^p<0.05; ^b^p<0.001.

**Supplementary Table 3:** Partial correlations between different biomarkers of kidney function

|  | **eGFR (ml/min/1.73m^2^)** |
| --- | --- |
| Urinary albumin (mg/L) **^ǂ^** | r= 0.03 |
|  | p=0.341 |
| UMOD (mg/creat) | **r= 0.11** |
|  | **p=0.002** |
| CKD273 classifier | **r= -0.22** |
|  | **p<0.001** |

Abbreviations: eGFR, estimated glomerular filtration rate; UMOD, uromodulin.

Notes: Adjusted for age, sex, and ethnicity.

**^ǂ^**  Urinary albumin additionally adjusted for urinary creatinine.

Bold values denote p<0.05.
